# Food-web stability signals critical transitions in temperate shallow lakes

**DOI:** 10.1038/ncomms8727

**Published:** 2015-07-15

**Authors:** Jan J. Kuiper, Cassandra van Altena, Peter C. de Ruiter, Luuk P. A. van Gerven, Jan H. Janse, Wolf M. Mooij

**Affiliations:** 1 Department of Aquatic Ecology, Netherlands Institute of Ecology, P.O. Box 50, 6700 AB Wageningen, The Netherlands.; 2 Aquatic Ecology and Water Quality Management Group, Department of Environmental Sciences, Wageningen University, P.O. Box 47, 6700 AA Wageningen, The Netherlands.; 3 Biometris, Wageningen University, P.O. Box 16, 6700 AC Wageningen, The Netherlands.; 4 Institute of Biodiversity and Ecosystem Dynamics, University of Amsterdam, P.O. Box 94248, 1090 GE Amsterdam, The Netherlands.; 5 PBL Netherlands Environmental Assessment Agency, P.O. Box 303, 3720 AH Bilthoven, The Netherlands.

## Abstract

A principal aim of ecologists is to identify critical levels of environmental change beyond which ecosystems undergo radical shifts in their functioning. Both food-web theory and alternative stable states theory provide fundamental clues to mechanisms conferring stability to natural systems. Yet, it is unclear how the concept of food-web stability is associated with the resilience of ecosystems susceptible to regime change. Here, we use a combination of food web and ecosystem modelling to show that impending catastrophic shifts in shallow lakes are preceded by a destabilizing reorganization of interaction strengths in the aquatic food web. Analysis of the intricate web of trophic interactions reveals that only few key interactions, involving zooplankton, diatoms and detritus, dictate the deterioration of food-web stability. Our study exposes a tight link between food-web dynamics and the dynamics of the whole ecosystem, implying that trophic organization may serve as an empirical indicator of ecosystem resilience.

## Abstract (web summary)

How mechanisms underlying food-web stability may influence ecosystem regime shifts is not well understood. Combining food-web and ecosystem modelling, Kuiper *et al*. show that destabilizing reorganization of a small number of key trophic interactions precede catastrophic changes in shallow lake ecosystems.

## Graphical abstract

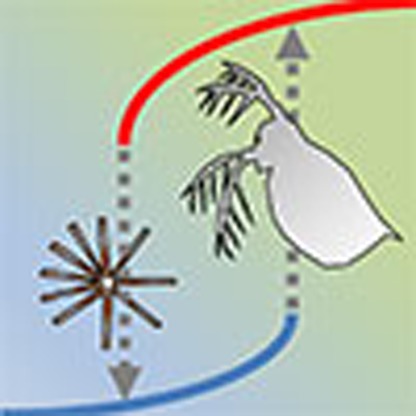

Current manifestations of anthropogenic stresses on ecosystems have intensified the need to understand and predict the resilience and stability of ecological systems^1^,^2^,^3^. Resilience and stability are topics that have inspired ecologists since the onset of the discipline^4^,^5^, and different theories and conceptual frameworks have developed around these topics, including alternative stable states theory and food-web theory.

Alternative stable states theory explains large scale catastrophic shifts in ecosystems—that is, the ultimate loss of resilience—from positive feedbacks and nonlinear interactions among biotic and abiotic key components of the system in relation to external forcings^6^,^7^,^8^. Catastrophic shifts are observed in various ecosystems including peatlands, rangelands, reef systems and shallow lakes, and generally occur unexpectedly^9^. Recent research has identified generic empirical indicators of resilience that might allow to anticipate critical transitions^9^.

Food-web theory elucidates which stabilizing mechanisms underlie the complex networks of trophic interactions that are found in nature, looking at the richness, patterning and strength of interactions among species^10^,^11^,^12^,^13^,^14^. As food webs reflect the flows of energy through a system, their features—including stabilizing properties—are important to ecosystem functions such as carbon and nutrient cycling^15^,^16^. Food webs provide an explicit link between community structure and the maintenance of ecosystem processes.

Although the conceptual frameworks of food webs and alternative stable states are highly influential in modern ecology, they developed independently and catastrophic regime shifts in ecosystems have seldom been explicitly linked to stability properties of complex trophic networks^17^. Here, we test whether indices for stability as defined by food-web theory can disclose an impending catastrophic shift in ecosystem state. On one hand, we hypothesize that food-web stability and ecosystem stability are inherently linked, considering the key role of food webs in governing the flows of energy through the ecosystem. On the other hand, we ask whether descriptions of food webs contain sufficient information on self-enhancing feedbacks to expose the nonlinear behaviour of the ecosystem in response to external forcing.

As a model system, we use temperate shallow lakes, for which abrupt changes between a submerged macrophyte-dominated state and a phytoplankton-dominated state are empirically well documented^18^,^19^. In this context, shallow lakes are particularly intriguing because many of the feedback loops that keep the system in each stable state involve the abiotic environment and are therefore not considered in a food-web approach to the system^6^.

We use a full-scale and well-tested dynamic ecosystem model of non-stratifying shallow lakes to simulate a catastrophic regime shift in ecosystem state. The model was originally developed to describe the main nutrient fluxes in Lake Loosdrecht in the Netherlands^20^,^21^, and has since been calibrated with data from more than 40 temperate lakes to obtain a best overall fit, making it suitable for more generalized studies on temperate shallow lakes^22^. The model has been successful in describing regime shifts in many case studies^23^.

We run the model for a range of nutrient loadings from oligotrophic to hypertrophic conditions and vice versa, to simulate the typical loading history of many shallow lakes in the temperate zone in the second half of the twentieth century^24^. For each loading level, we run the model until the seasonally forced equilibrium is reached, and obtain the average chlorophyll-*a* concentration to characterize the state of the lake ecosystem; chlorophyll-*a* is one of the most common proxies for water quality used by ecosystem managers. Also, we collect food-web data from the ecosystem model to construct material flux descriptions of the aquatic food web at each loading level (Fig. 1)^25^,^26^.

From these food-web properties, we estimate the per capita interaction strengths between the trophic groups, using established methods typically used by food-web ecologists to describe empirical food webs^11^,^13^, based on the principles of May^10^ and Lotka-Volterra type equations^11^,^26^. Interaction strengths represent the size of the effects of species on each other's dynamics near equilibrium and define the elements of the (Jacobian) community matrix representation of the food web^10^. Food-web stability is assessed using the diagonal strength metric (*s*)^27^,^28^, being the minimum degree of relative intraspecific interaction needed for matrix stability. Thus, for each level of nutrient loading, we obtain a parameterized (Jacobian) community matrix description of the food web embedded in the ecosystem and evaluate its stability.

The results of this combined modelling approach show that imminent shifts in ecosystem state during eutrophication and re-oligotrophication are preceded by a destabilizing reorganization of the trophic web. This suggests that trophic organization can serve as an empirical indicator of ecosystem resilience. We show that only few key trophic interactions dictate the decrease of food-web stability, particularly among lower trophic level groups, and emphasize the role of destabilizing trophic cascades. Hence, by using a food-web approach to ecosystem stability, we refine our mechanistic understanding of the biological processes underlying the sudden shifts in ecosystem state.

## Results

### Ecosystem response to nutrient loading

**Figure 2:**
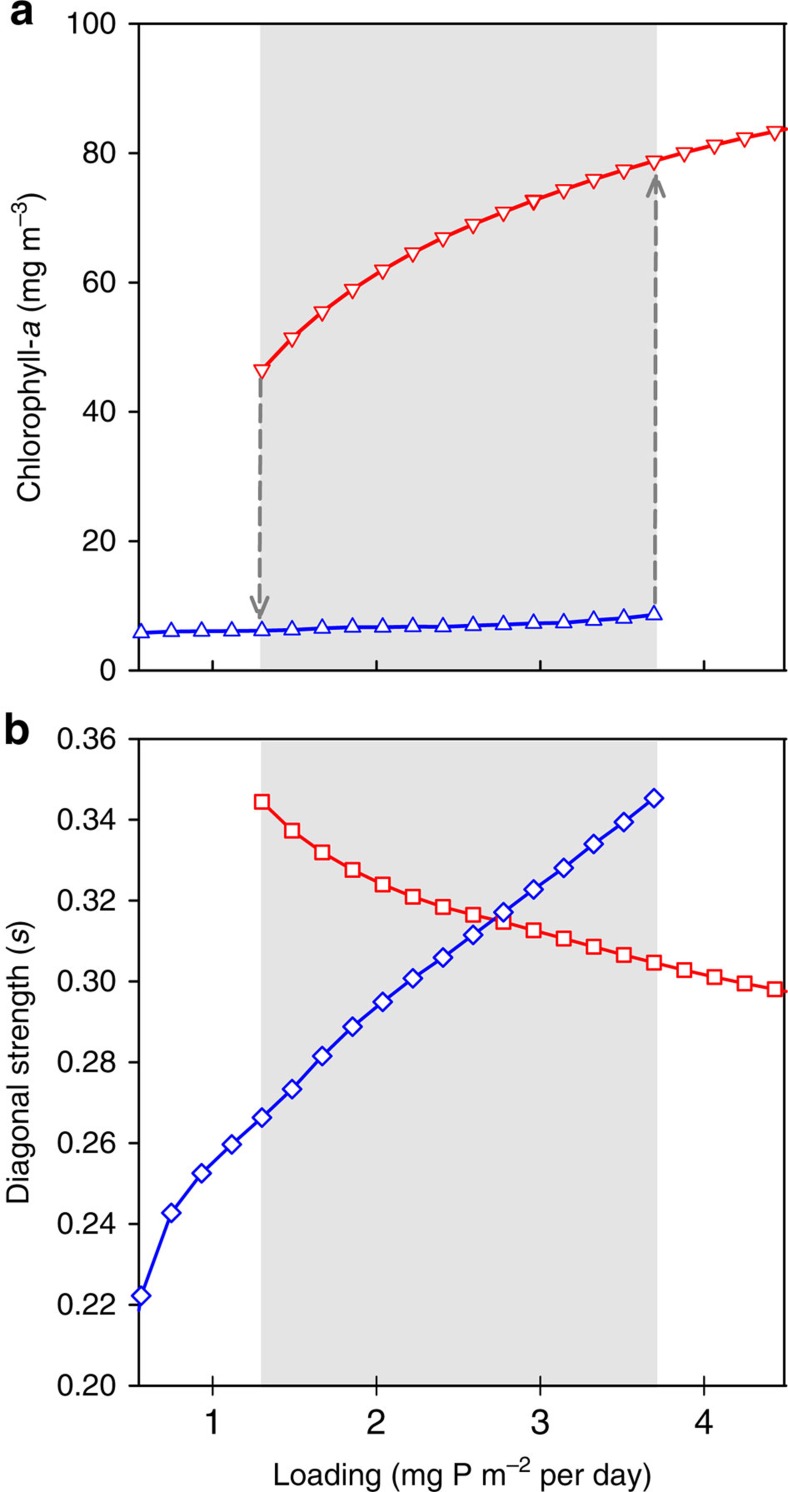
Ecosystem and food-web response to nutrient loading. (**a**) The equilibrium concentration (yearly average) chlorophyll-*a* in the water column, as proxy for the ecosystem state, for two initial states: a clear- (blue upward triangles) and a turbid-water state (red downward triangles). (**b**) Food-web stability, represented by the intraspecific interaction needed for matrix stability (*s*) for food webs in a clear- (blue diamonds) and a turbid-water state (red squares). Stability decreases with increasing *s*.

The bifurcation analysis of the full-scale shallow lake ecosystem model showed the occurrence of alternative stable states between a phosphorus (P) loading of 1.3 and 3.7 mg P m^−2^ per day (Fig. 2a). During eutrophication (Fig. 2a, blue line), the macrophyte-dominated clear-water state marked by a low level of chlorophyll-*a* disintegrates abruptly when the critical phosphorus loading is reached, shifting the system to a phytoplankton-dominated state with high levels of chlorophyll-*a*. During re-oligotrophication (Fig. 2a, red line), the system lingers in the turbid state until the phosphorus loading is much reduced and the reverse shift back to the clear-water state occurs. The delayed response of chlorophyll-*a* to changes in nutrient loading—that is, hysteresis—is consistent with many field observations, which provide strong empirical evidence for the existence of alternative stable states^18^,^29^. An important observation here is that in the clear-water state, the average chlorophyll-*a* level hardly responds to eutrophication (Fig. 2a), and thus gives no indication for the loss of resilience in the system.

### Food-web response to nutrient loading

We followed the interaction strengths in the trophic web and evaluated food-web stability along the eutrophication axis using diagonal strength as an indicator (see Methods). We found that with increasing lake productivity (Fig. 2b, blue line), destabilizing changes in the food web occurred: decreasing food-web stability forebodes the catastrophic shift. This result is not trivial because the ecosystem model and the food-web model differ distinctly in structure and shape of the interactions. At the critical nutrient loading, the food web underwent a drastic reorganization to a phytoplankton-dominated configuration, coinciding with a sudden increase in stability (decrease in diagonal strength, from blue to red line in Fig. 2b). Intriguingly, we found that during re-oligotrophication (Fig. 2b, red line), which is needed for ecosystem recovery, a similar decrease in food-web stability was visible, again followed by a sudden re-establishment of stability once the critical nutrient loading for ecosystem recovery was reached. Thus, depending on the trophic organization of the food web, enrichment and impoverishment can both be destabilizing, even though the topology of the web is the same. From an alternative stable states point of view, this can be explained as clear- and turbid-water states each having a basin of attraction that deteriorates towards a tipping point. Hence, we find food-web stability to be associated with the resilience of the attracting equilibrium.

### Identifying stabilizing and destabilizing interactions

Food-web stability is an aggregated measure with a multitude of underlying processes. We here present an innovative approach to decipher which interactions are primarily responsible for the eroding stability during eutrophication and re-oligotrophication. At a given level of nutrient loading, the stability metric *s* follows directly from the interaction terms in the (Jacobian) community matrix. By varying the strength of each element in the matrix, we calculated the relative sensitivity of *s* to changes in each specific trophic interaction: 
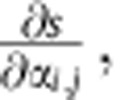
 where *α*_*i,j*_ is the interaction effect of species *j* on species *i*. As such, we reveal the intrinsic dynamics of the food web, that is, how stability is constrained by the architecture of the food web. Besides the sensitivity, the effect of *α*_*i,j*_ on *s* depends on the actual change of *α*_*i,j*_ in response to nutrient loading 
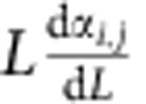
. Note that changes in interaction strength along the nutrient loading axis may be imposed by forces in the ecosystem that are not explicitly considered in the food-web model, such as oxygen dynamics and stoichiometry. Taken together, the following formula can be used to disentangle which and how changing interactions contribute to the weakening of stability (Supplementary Fig. 1):


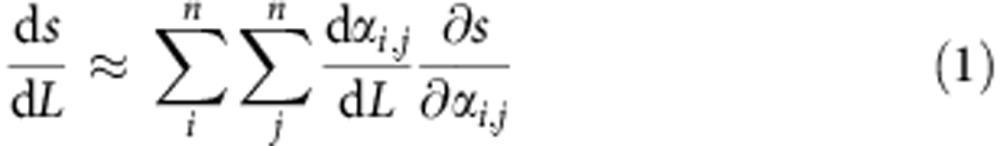


**Figure 3:**
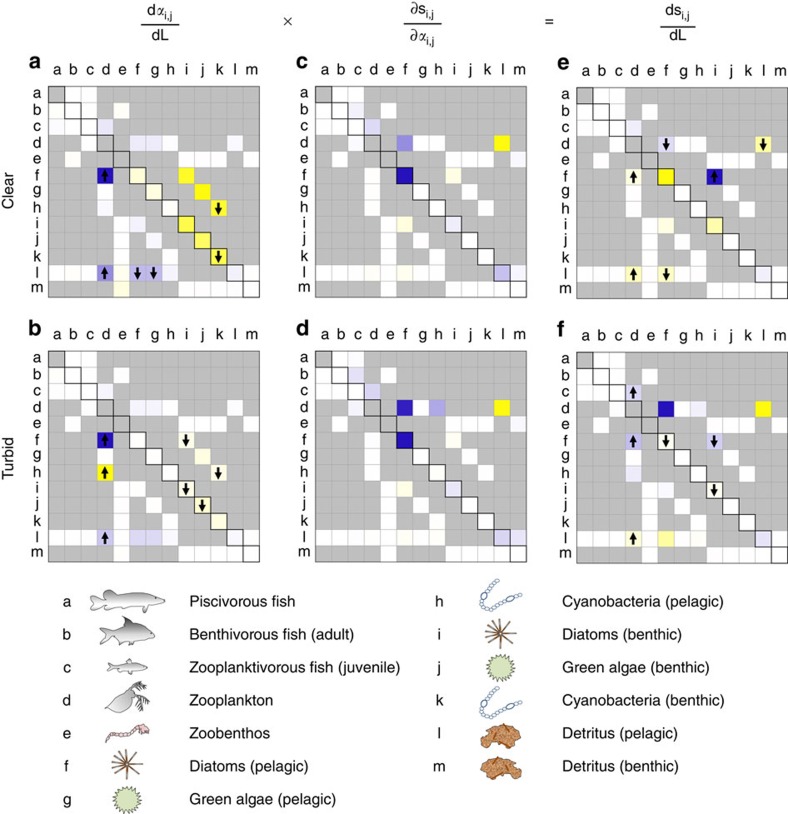
Graphical summarization of the changing trophic interactions and their impact on food-web stability. The left panels show which interaction terms are impacted by changing nutrient loading. Cell colour indicates whether interaction strength increases (blue), decreases (yellow) or does not change (white) during eutrophication (**a**) and re-oligotrophication (**b**). Colour intensity depicts the relative magnitude of change. Arrows indicate whether the change is notably progressive (upward) or descending (downward) towards the regime shift. The middle panels (**c**,**d**) show the sensitivity of food-web stability to changes in interaction strengths. An increase of interaction strength can have a positive effect (blue cells), negative effect (yellow cells) or no effect (white cells) on stability (and hence an inverse effect on *s*). The intensity of the colour indicates the relative magnitude of the effect. The right panels show the contribution of each interaction term to the impact of eutrophication (**e**) and re-oligotrophication (**f**) on food-web stability, which is the product of the foregoing. Colours indicate whether interactions have a positive (blue), negative (yellow) or no effect (white) on stability (and inversely on *s*).

We found that both during eutrophication (Fig. 3a) and re-oligotrophication (Fig. 3b), several interactions in the lake food web increased or decreased in strength in response to changing nutrient loading. The majority of these interactions involved zooplankton, benthic and pelagic phytoplankton species or detritus. Most interactions, however, were unaffected by changing nutrient loading. When we analysed the sensitivity of food-web stability to changes in specific interaction strengths, we found that food-web stability is sensitive to only a select number of interactions, and that there is just a partial overlap with the interactions that actually changed along the loading axes (Fig. 3c,d). As a result, the observed changes in food-web stability during eutrophication and re-oligotrophication can be attributed to only a handful of interactions, involving detritus, diatoms and zooplankton (Fig. 3e,f). The strengths of these interactions change along the eutrophication axis, and the food-web stability is sensitive to these interactions. Most destabilizing were the interaction effects between zooplankton and detritus, the effect of pelagic diatoms on detritus and the effect of pelagic diatoms on themselves relating to sedimentation (Fig. 3, Supplementary Fig. 2).

**Figure 4:**
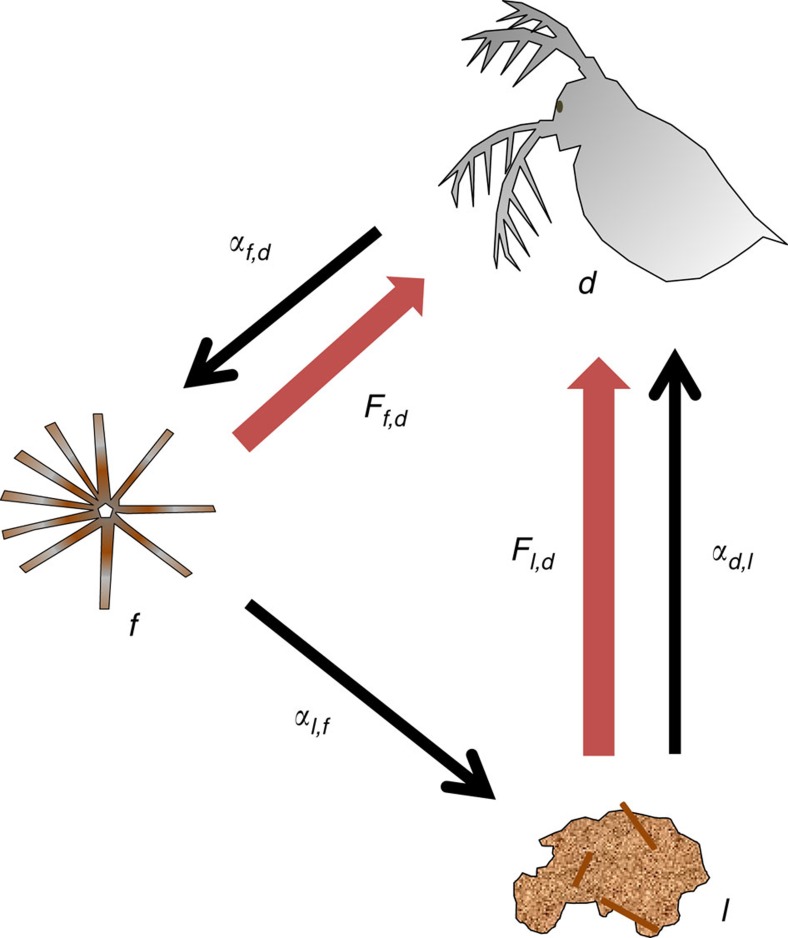
Loop with the heaviest loop weight. The omnivorous three link loop with zooplankton (*d*), pelagic diatoms (*f*) and pelagic detritus (*l*) is the heaviest loop in the trophic network. Black arrows indicate the direction of the interaction effect (*α*). Red arrows indicate the feeding fluxes (*F*). The top-down effect of zooplankton on diatoms is a negative effect directly resulting from consumption. The effect of diatoms on detritus results from natural mortality of diatoms and the unassimilated part of diatom consumption by zooplankton. The bottom-up effect of detritus on zooplankton is a positive predation effect.

**Figure 5:**
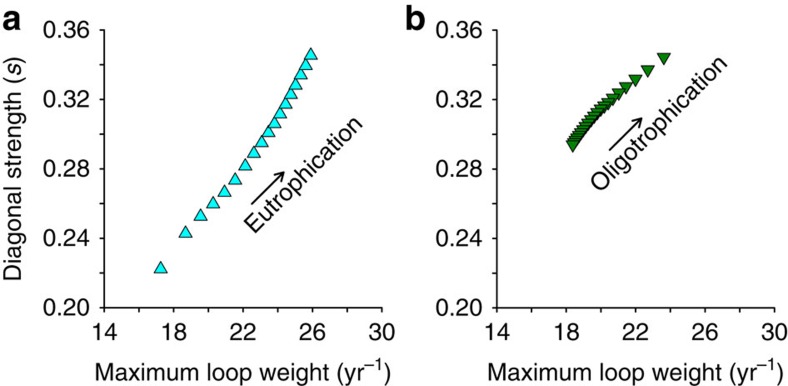
Stability versus maximum loop weight. The maximum loop weight (per year (yr^−1^)) shows a positive relationship with intraspecific interaction needed for matrix stability (s) during (**a**) eutrophication and (**b**) re-oligotrophication. Food-web stability decreases with increasing *s*.

We supported these results by calculating the loop weights of all the ‘trophic interaction loops' in the trophic web along the nutrient loading axis (see Methods)^27^. We found that, under all conditions, the loop with the highest weight, which is considered the Achilles heel of a trophic network^13^, was the omnivorous loop that linked the same three groups: detritus, diatoms and zooplankton (Fig. 4). The maximum loop weight increased towards both regime shifts, from either direction of nutrient loading, and was strongly correlated to the amount of intraspecific interaction needed for matrix stability^27^ (Fig. 5).

We analysed the biomasses and feeding rates underlying the interactions in the trophic interaction loop that has the maximum weight to disentangle what caused the increase of the loop weight (Fig. 4, Table 1). We observed that, during eutrophication, the feeding rates increased relatively more than the biomasses. As interaction strengths depend largely on the ratio of feeding rate to population densities (see Methods), this pattern led to an increase in interactions strengths, and hence, in a higher loop weight. Particularly, the increase in the interaction effect of detritus on zooplankton, which is the weakest interaction in the loop, contributed to the enhancement of the loop weight (Table 1). The regime shift to the turbid cyanobacteria-dominated state resulted in an unfavourable climate for zooplankton as their biomass was reduced. The conditions for zooplankton improved however during re-oligotrophication as we observed increasing feeding rates towards the regime shift. The biomasses of the trophic groups were only moderately affected by the reduction of nutrient loading, wherefore the interaction strengths increased along this axis. This time, the increase in loop weight was dictated by the effect of zooplankton on diatoms, as the feeding on diatoms increased more than the feeding on detritus (Table 1).

## Discussion

Our results show that a decrease in ecosystem stability coincides with a decrease of food-web stability, which supports the prevailing view in food-web ecology that non-random patterns of strong and weak trophic interactions confer stability to the ecosystem level^30^.

From an alternative stable state perspective, it may seem surprising that food-web metrics can reveal the impending shift without explicitly including the feedbacks through the abiotic environment that are thought to be crucial for regime shifts in lakes, such as shading, provision of refugia and retention of P in the sediment^6^. We resolve this by realizing that the observed webs at each level of nutrient loading are shaped by forces that are not part of the food-web model *per se*, implicitly carried over to the food-web model during sampling of the food-web data. Using expression 1, we made a clear distinction between the intrinsic dynamical properties of the food web 
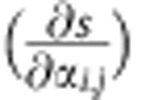
 and the changes in interaction strengths driven by the changing nutrient loading to the ecosystem 
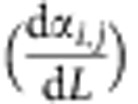
.

Equivalently interesting is that the weakening of stability is exposed without explicitly taking nonlinear interaction terms into account, as relatively simple Lotka-Volterra dynamics underlie the computation of food-web stability. The use of linear interaction terms in food-web models greatly eases the estimation of interaction strengths from empirical data^26^,^31^, but has implications for the stability properties of dynamical systems^32^, potentially hampering a one-to-one mathematical transfer of stability properties from the ecosystem to the food-web model. Nonetheless, Lotka-Volterra dynamics has been used in numerous studies to describe empirical food webs and disclose stabilizing patterns of strong and weak links^11^,^13^,^33^, and there is mounting experimental evidence that the exposed patterns indeed confer stability to the level of communities^30^ and ecosystem processes^34^. It appears that the importance of the patterning of strong and weak trophic links in ecosystems overshadows that of the exact shape of the functional response used to describe the interactions.

Our analyses reveal that only few trophic interactions dictate the deterioration of food-web stability, particularly among zooplankton, diatoms and detritus. This is in line with empirical studies on interaction strengths suggesting that most interactions have only a negligible impact on community dynamics^11^, and is consistent with alternative stable states theory that regime shifts in ecosystems can be explained from only few key components in relation to external forcing^7^. The interplay between zooplankton and phytoplankton has often been claimed to be pivotal in controlling aquatic ecosystem dynamics and causing alternative stable states^35^.

Zooming in on the interactions that correlated most with stability exposed a destabilizing trophic cascade during eutrophication and re-oligotrophication. In the clear-water state, the ratio of feeding rate to predator biomass increased with productivity through a classic trophic cascade^36^,^37^, which resulted in a destabilizing increase of interaction strengths, and hence, a negative productivity–stability relationship. Somewhat paradoxically, another destabilizing trophic cascade occurred during re-oligotrophication, even though the overall productivity was decreasing. A shift in phytoplankton dominance enhanced the trophic transfer efficiency, resulting in an increase in destabilizing interaction strengths. This pattern of shifting dominance during re-oligotrophication, to the detriment of cyanobacteria and the benefit of more edible diatoms and green algae, is consistent with field observations^38^.

Our finding that most interactions have only a negligible impact on community dynamics does not imply that species are redundant, as extreme changes in interaction strength—for example, owing to species extinctions—can have strong nonlinear effects on community stability. A next step will be to investigate the synergetic effects of food-web manipulations and environmental stress, as it is unquestionable that species extinctions and invasions can have far-reaching consequences for ecosystem functioning, of which the introduction of the Nile perch to the world's second largest freshwater system Lake Victoria gives one of the most striking examples^39^.

Our results indicate that food-web stability can be used as an empirical indicator of ecosystem resilience. The established food-web methods that we used can be turned into a tool for managers to evaluate food-web stability on a yearly basis. Food-web stability as an early warning signal is of a fundamentally different nature than the conventionally used critical slowing down or flickering^9^. Instead, the method is more akin to an alternative generalized modelling approach recently proposed^40^, which has the potential advantage of being less dependent on high resolution time series^41^. Many of the limitations that have been identified for conventional early warning signals also apply to food-web stability^41^. For example, food-web stability gives no information about the distance to a regime shift, and needs a baseline to be meaningful. To overcome such limitations, it has been suggested that the combined use of several independent indicators is needed to confidently disclose an impending regime shift^42^. Food-web stability can be a valuable addition to the current set of indicators in this respect. We anticipate that palaeolimnological reconstructions of food webs^43^, and microcosm experiments with multiple nutrient treatments^44^, are needed to uncover the true potential and practical limitations of this early warning signal, such as sensitivity to false alarms^41^.

By showing that food-web stability signals critical transitions in a shallow lake ecosystem, we reconcile the conceptual frameworks of food webs and alternative stable states. The food-web stability approach laid out here opens up ways to obtain a better mechanistic understanding of the biological processes underlying sudden shifts in the ecosystem state, bringing us closer to providing a sound mechanistic basis for predicting ecosystem dynamics in a changing world^45^.

## Methods

### Ecosystem modelling

We used a well-established integrated dynamical model for shallow lakes—PCLake—to simulate a critical transition of a shallow non-stratifying lake^22^. The model embraces several key ecological concepts including closed cycles of nutrients and matter, benthic-pelagic coupling, stoichiometry, food-web dynamics and trophic cascade. The aquatic food web is modelled on the basis of functional groups and comprises four trophic layers. The pelagic and benthic food chains are coupled via a shared predator, reproduction of fish and the settling and resuspension of detritus and phytoplankton.

The model has been calibrated against the data of >40 lakes resulting in lake characteristics resembling an ‘average' shallow lake in the temperate zone^22^. We used default parameter settings, describing a lake with a mean depth of 2 m, a fetch of 1,000 m, a water inflow of 20 mm per day, a lightly clayish soil and no wetland zone, and initial values for two contrasting ecosystem states (clear versus turbid)^22^.

We ran the model for various phosphorus (P) loadings in the range of 0.1 to 5 mg P m^−2^ per day in steps of 0.18, starting with either an initially clear- or an initially turbid-water state. The nitrogen loading was consistently kept 10 times the P loading to maintain phosphorus limitation. For each loading, the model was run for 20 years to reach seasonally forced equilibrium conditions. Output data of the final year was used to characterize the state of the ecosystem and to compile material flow descriptions of the food web using established food-web methods (see below). A more detailed description of the ecosystem model, and the bifurcation analysis with nutrient loading, can be found in ref. 22 and references therein.

### Material flow descriptions

For each nutrient loading level, we constructed material flow descriptions of the corresponding food web, following a typical food-web approach as presented by ref. 25 and ref. 26. We calculated feeding rates, flows to the detritus pools and reproduction rates from yearly average biomass densities, death rates, prey preferences and energy conversion efficiencies, which we extracted from the ecosystem model. Assuming steady state and the conservation of matter, the production of each population must balance the rate of loss through natural mortality and predation: 
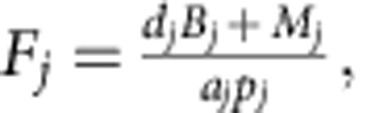
 where *F*_*j*_ is the feeding rate (g m^−2^ per year) of species *j*, *d*_*j*_ is the specific death rate (per year), *B*_*j*_ is the average population density (g m^−2^), *M*_*j*_ is the mortality by predation (g m^−2^ per year), *a*_*j*_ is the assimilation efficiency and *p*_*j*_ is the production efficiency (both dimensionless). For the juvenile (zooplanktivorous) fish and adult (benthivorous) fish, the reproduction fluxes were added to the numerator. When a predator feeds on several trophic groups, the prey preferences were included to calculate the feeding rate of predator *j* on prey species *i*:
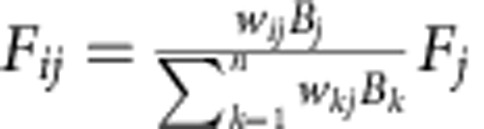
, where *w*_*ij*_ refers to the preference of predator *j* for prey *i*, and *n* is the number of prey types. The fluxes arising from natural mortality go to the detritus pools, just as the unassimilated fraction of the feeding rate (1−*a*_*j*_*)·F*_*ij*,_ representing the biomass that is not actually consumed or is egested. Calculations started at the top of the food chain, as the top predator does not experience predation. The values of the parameters are listed in Supplementary Table 1. The parameters are assumed constant for all the nutrient loadings. The settling and resuspension rates of detritus and phytoplankton (g m^−2^ per year) were directly extracted from the ecosystem model. Macrophytes are not consumed directly but as detritus and are therefore only considered as input for the detritus pools.

### Food-web dynamics

We developed a Lotka-Volterra type food-web model that included the same trophic groups as the full ecosystem model, in the form 
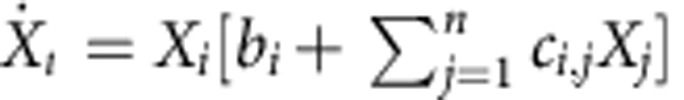
 and extensions thereof, where *X*_*i*_ and *X*_*j*_ represent the population sizes of groups *i* and *j*, *b*_*i*_ is specific rate of increase or decrease of group *i*, and *c*_*ij*_ is the coefficient of interaction between group *i* and group *j*. Interaction strengths can be defined as the partial derivatives of Lotka-Volterra type growth equations in equilibrium and give the elements of the (Jacobian) community matrix representation of our model^10^. The interaction effect of predator *j* on prey *i* can be expressed as 
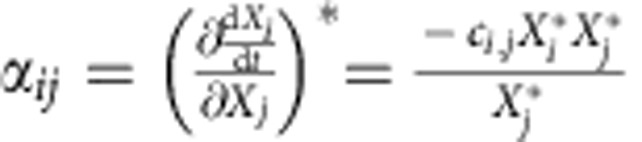
 (a detailed description of all the equations can be found in Supplementary Note 1).

The values of the partial derivatives can be directly derived from the material flow descriptions of the food web, using the criterion developed by May^10^,^11^. Here, the assumption is that the average annual feeding rate *F*_*i,j*_ (g m^−2^ per year) can be expressed as *−c*_*i,j*_*X*_i_^*^X_j_^*^, that is, the death rate of group *i* due to predation by group *j* in equilibrium^11^. Thus, the strength of this interaction can be derived by dividing the feeding rate by the annual average population density of the predator 
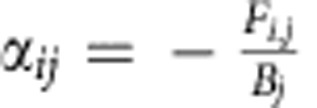
. The opposite (positive) effect of the prey on the predator, as well as the interaction terms resulting from the detrital fluxes, reproduction fluxes and settling and resuspension fluxes, were determined in a similar way^26^ (see Supplementary Note 1).

We calculated interaction strengths and constructed (Jacobian) community matrices from the material flow descriptions of the food webs at each loading level for each initial state. A randomization procedure confirmed that the imposed patterns of interaction strengths were non-random, and thus crucial to the stability of the food web (Supplementary Fig. 3)^11^,^27^.

### Calculation of stability

For the consumers and the phytoplankton groups in the food web, we assume that, for equilibrium conditions, the death rate *d*_*i*_ (per year) can be split in density-independent death, and density-dependent death: *d*_*i*_*=(*1−*s)d*_*i*_*+sd*_*i*_, where *s* represents the fraction of the death rate *d*_*i*_ caused by density-dependent mortality (per year). When taking the partial derivatives of the differential equations to determine the (Jacobian) community matrix, this *s* will occur on the diagonal of the matrix, representing intraspecific interaction strengths *α*_*ii*_=−*s.d*_*i*_. We followed Neutel *et al*.^13^,^27^ and measured stability as the minimum degree of relative intraspecific interaction needed for matrix stability (all eigenvalues having negative real parts), assuming the same value for *s* for all trophic groups. Food webs that need less intraspecific interference (a smaller value for *s*) are more stable. There is a close relation between *s* and the dominant eigenvalue of a matrix without added intraspecific interference (Supplementary Fig. 4). The use of *s* however has the advantage of providing a biological interpretation of stability^13^.

### Calculation of the maximum loop weight

The weight of a trophic feedback loop—a closed chain of trophic links—is defined as the geometric mean of the absolute values of the interaction strengths that compose the loop^13^,^27^: 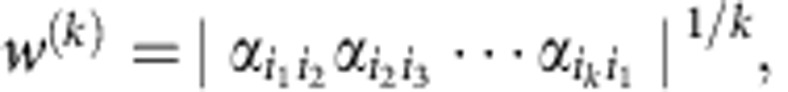 where *k* is the number of species in the loop. The maximum loop weight gives an approximation of the level of intraspecific interference needed for matrix stability^27^.

## Additional information

**How to cite this article:** Kuiper, J.J. *et al*. Food-web stability signals critical transitions in temperate shallow lakes. *Nat. Commun.* 6:7727 doi: 10.1038/ncomms8727 (2015).

## Supplementary Material

Supplementary Information Supplementary Figures 1-4, Supplementary Table 1, Supplementary Note 1 and Supplementary References

## Figures and Tables

**Figure 1:**
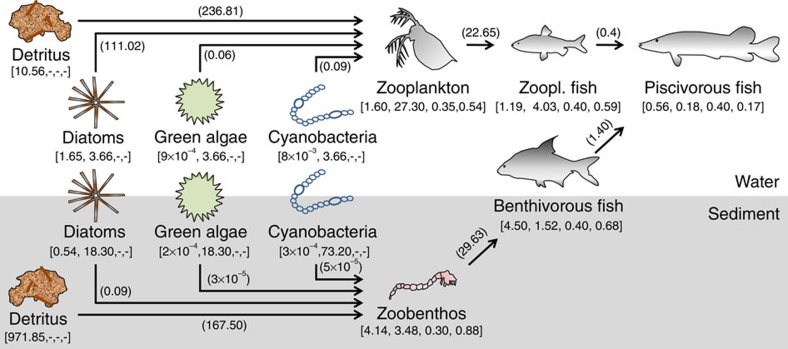
Schematic representation of the aquatic food web and the feeding relations. The food web comprises a pelagic and benthic food chain linked by a shared predator. Data (square brackets) used to calculate feeding rates (parentheses) are given in the sequence biomass (g m^−2^), specific death rate (per year), assimilation efficiency and production efficiency. Feeding rates (g m^−2^ per year) are given near their respective arrows. Settling, resuspension and reproduction fluxes and flows to the detritus pools are not represented here but were included in the analyses. The data belong to a clear-water state receiving 2.6 mg P m^−2^ d^−1^.

**Table 1:** Building blocks of the heaviest loop at different nutrient loadings.

**Property**	Loading (mg P m^−2^ per day)			
	Eutrophication		Re-oligotrophication	
	0.5	3.5	4.8	1.3
Loop weight (per year)	17.25	25.90	18.46	23.62
				
Biomasses (g m^−2^)				
* *Zooplankton, *d*	0.94	1.61	1.18	1.11
* *Diatoms (pelagic), *f*	1.41	1.87	3.43	3.53
* *Detritus (pelagic), *l*	6.44	10.89	11.15	9.84
				
Feeding rate (g m^−2^ per year)				
* F*_*f,d*_	58.97	128.62	122.26	157.40
* F*_*l,d*_	89.89	249.35	132.31	146.41
* F*_total_	148.89	386.85	321.11	344.91
				
Interaction strengths (per year)				
* α*_*f,d*_	−62.60	−79.68	−103.77	−142.40
* α*_*l,f*_	30.87	48.33	26.81	32.68
* α*_*d,l*_	2.66	4.36	2.26	2.83

The loop weight is calculated from the interaction strengths: *w=|α*_*f,d*_·*α*_*l,f*_·*α*_*d,l*_*|*^1/3^.

Besides rates of the feeding of zooplankton on diatoms and detritus, the total feeding rate of zooplankton is presented, also comprising the feeding on green algae and cyanobacteria.
