# Supplementary material for: Food-web stability signals critical transitions in temperate shallow lakes
Source: Nat Commun. 2015 Jul 15;6:7727. doi: 10.1038/ncomms8727 (PMC4518252; doi:10.1038/ncomms8727)
Supplement: Supplementary Information — Supplementary Figures 1-4, Supplementary Table 1, Supplementary Note 1 and Supplementary References [file ncomms8727-s1.pdf]

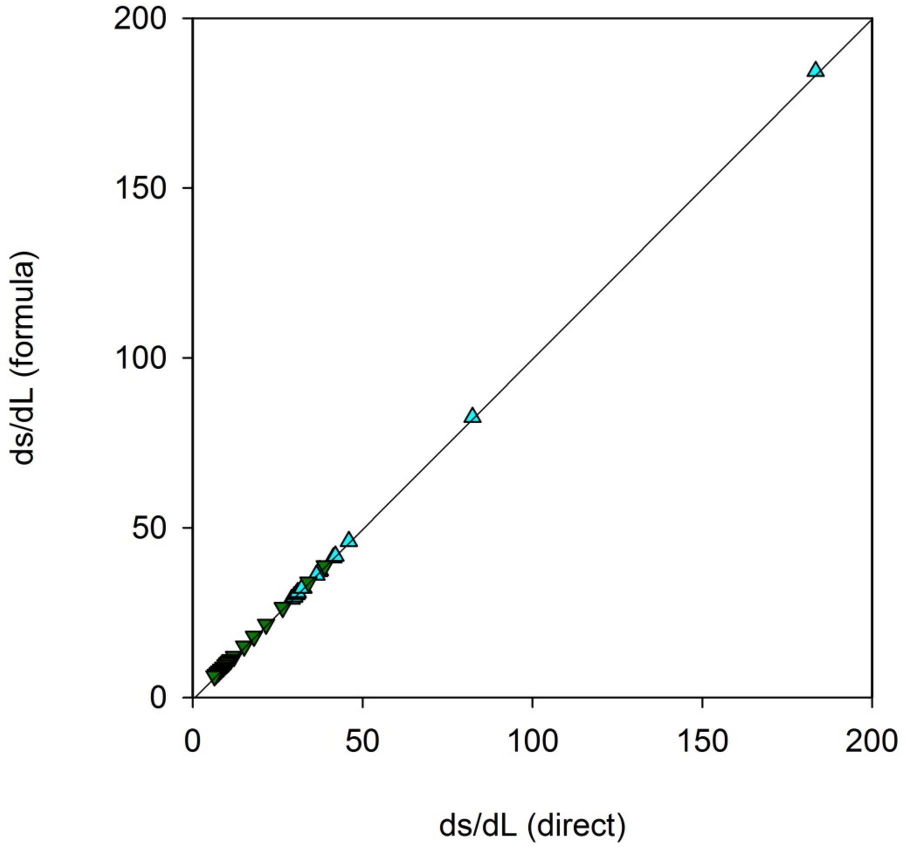

Supplementary Figure 1. Proof of concept of expression 1. We show the comparison of the change in matrix stability per unit nutrient loading ($\frac{ds}{dL}$) with the sum of the multiplications of the changes in strength and the sensitivities of all individual interactions in the matrix $(\sum_{i}^{n}\sum_{j}^{n}\frac{d\alpha_{i,j}}{dL}\frac{\partial s}{\partial \alpha_{i,j}})$. We plotted the relationship for each step along the loading axis from clear to turbid (turquoise upward triangles), and from turbid to clear conditions (dark green downward triangles).

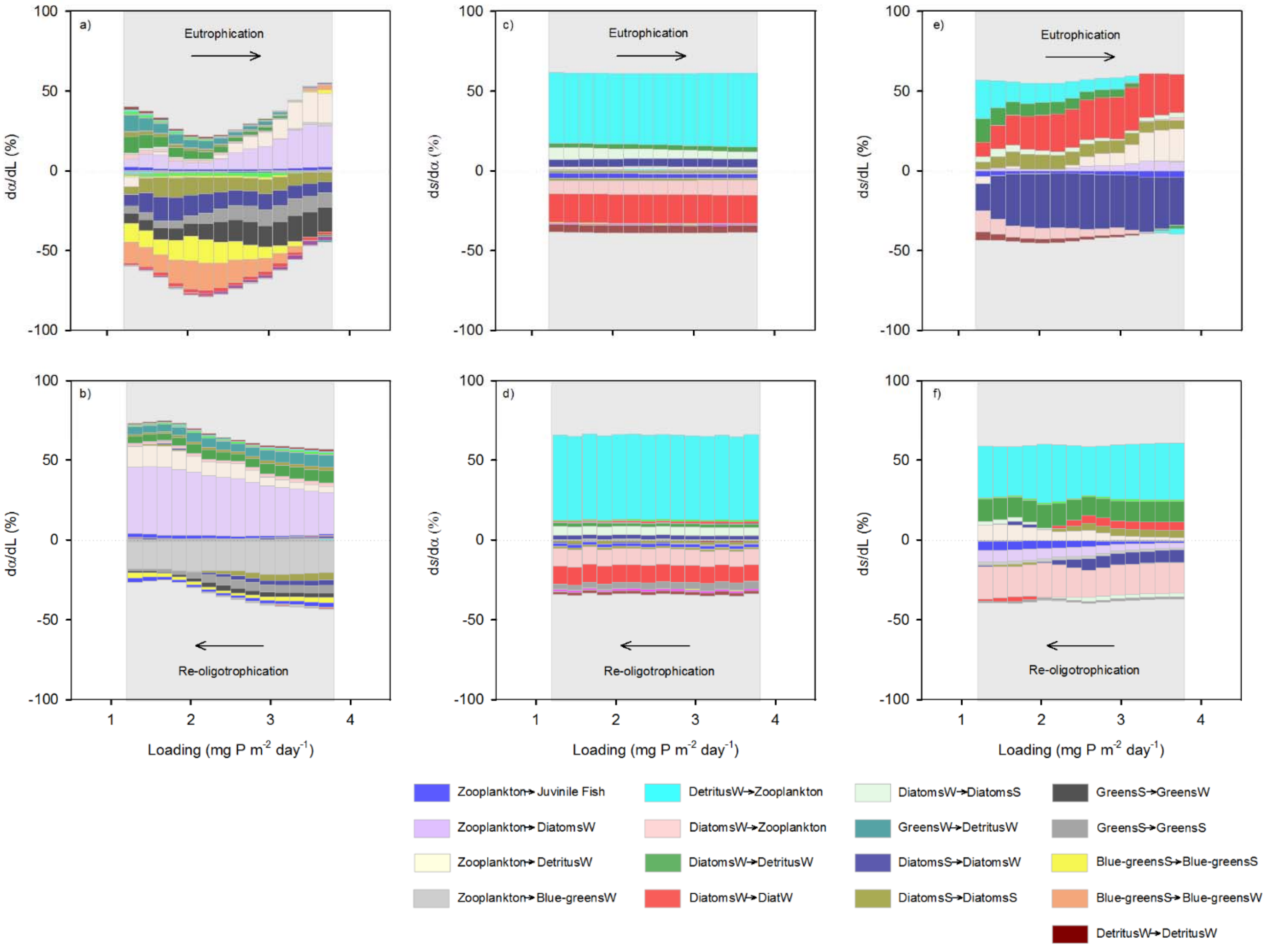

Supplementary Figure 2. Elucidating which interactions contribute to the decrease of food-web stability. The response of food-web stability to nutrient loading depends both on how the strengths of trophic interactions change in response to nutrient loading: $d\alpha_{i,j}/dL$, and the extent to which the stability of the food web is sensitive to those changes: $\partial s/\partial\alpha_{i,j}$. Plotting the relative changes of the interaction strengths per unit change of loading L during eutrophication **(a)** and re-oligotrophication **(b)** reveals that several of the 55 interactions in the food web increase or decrease in strength towards the tipping point. A positive change indicates that the strength of the interaction increases and a negative change indicates that the strength of the interaction is decreasing. Plotting the relative sensitivity of the diagonal strength s to changes in the interaction strengths during eutrophication **(c)** and re-

oligotrophication **(d)** reveals that the stability is only sensitive to a select number of interactions, and that the sensitivity is not dependent on the location along the loading axis. The product of the change in strength of each interaction with the sensitivity of *s* to that interaction strength gives the relative contribution of each interaction to changes in *s*, during eutrophication **(e)** and re-oligotrophication **(f)**. Only changes in a handful of stabilizing and destabilizing interactions contribute to changes in stability - mainly involving detritus, diatoms and zooplankton. Only relevant interactions are presented in the legend. Phytoplankton species and detritus may appear both in the pelagic and in the sediment layer: they are abbreviated and indicated with 'W' if they are in the pelagic, or 'S' if they are found in or on the sediment.

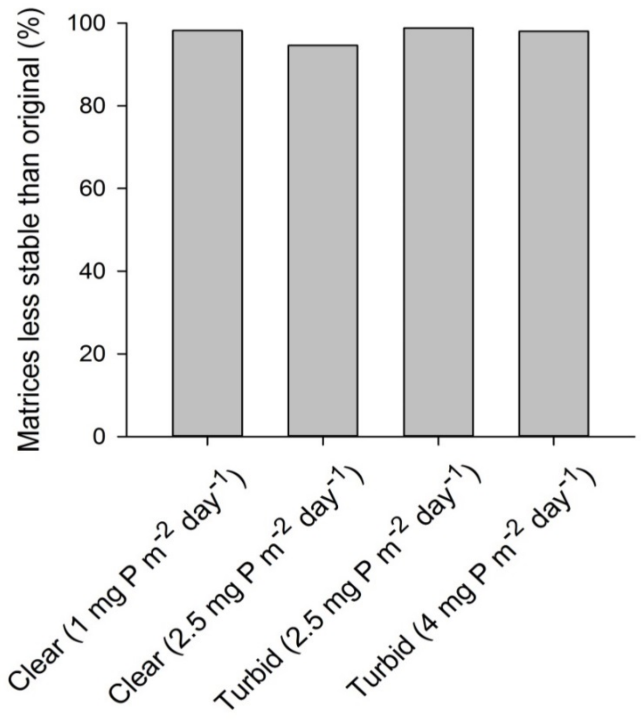

Supplementary Figure 3. Percentage of randomized matrices less stable than original (Jacobian) community matrix representation of the aquatic food web. We randomized the matrices of four lakes differing in their initial conditions or nutrient loading 500 times and compared the stability of the randomized matrices with the stability of the original matrices. Randomization was performed by randomly exchanging pairs of interaction strengths but keeping the pairs as such intact, preserving both the sign structure of the matrix and the overall strength of the trophic interactions relative to the strength of intragroup interference: the randomized matrices have thus a similar structure but lost the pattern that resulted from the ecosystem model.

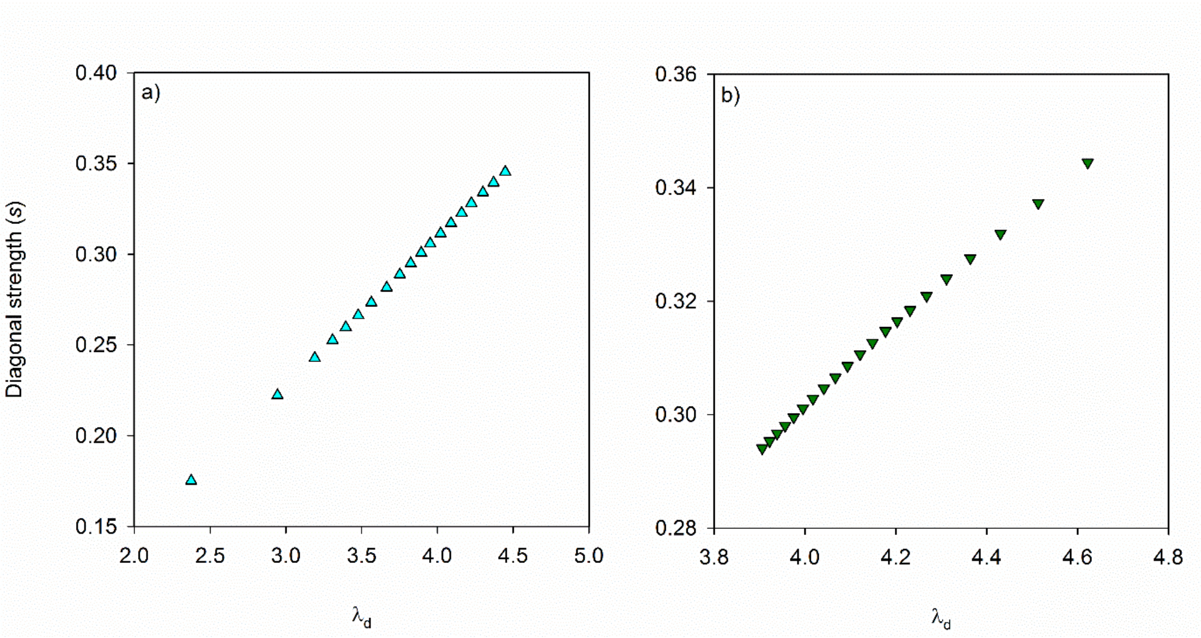

Supplementary Figure 4. Relation between the maximum eigenvalue and the relative intraspecific competition needed for matrix stability. The relation between the maximum eigenvalue λ_d and the relative intraspecific competition s is plotted for each step along the loading axis from **(a)** clear to turbid, and **(b)** from turbid to clear conditions. Unlike the maximum eigenvalue, the level of intraspecific interaction strength s has a biological interpretation as it translates to a loss rate of organisms at steady state relative to the total loss that the system can provide for.

Supplementary Table 1: Overview of the parameters and output of the ecosystem model PCLake used to estimate material fluxes and interaction strengths in the food web.

Class	Trophic group	Unit	Value	Source
Biomass	Piscivorous fish	g m^{-2}	Variable	PCLake simulation – yearly average
Biomass	Zoopl. fish (juvenile)	g m^{-2}	Variable	PCLake simulation – yearly average
Biomass	Benth. fish (adult)	g m^{-2}	Variable	PCLake simulation – yearly average
Biomass	Zooplankton	g m^{-2}	Variable	PCLake simulation – yearly average
Biomass	Benthos	g m^{-2}	Variable	PCLake simulation – yearly average
Biomass	Pelagic Diatoms	g m^{-2}	Variable	PCLake simulation – yearly average
Biomass	Pelagic Green algae	g m^{-2}	Variable	PCLake simulation – yearly average
Biomass	Pelagic Cyanobacteria	g m^{-2}	Variable	PCLake simulation – yearly average
Biomass	Pelagic Detritus	g m^{-2}	Variable	PCLake simulation – yearly average
Biomass	Benthic Diatoms	g m^{-2}	Variable	PCLake simulation – yearly average
Biomass	Benthic Green algae	g m^{-2}	Variable	PCLake simulation – yearly average
Biomass	Benthic Cyanobacteria	g m^{-2}	Variable	PCLake simulation – yearly average
Biomass	Benthic Detritus	g m^{-2}	Variable	PCLake simulation – yearly average
Settling flux	Pelagic Diatoms	g m^{-2} $year^{-1}$	Variable	PCLake simulation – year summation
Settling flux	Pelagic Green algae	g m^{-2} $year^{-1}$	Variable	PCLake simulation – year summation
Settling flux	Pelagic Cyanobacteria	g m^{-2} $year^{-1}$	Variable	PCLake simulation – year summation
Settling flux	Pelagic Detritus	g m^{-2} $year^{-1}$	Variable	PCLake simulation – year summation
Resuspension flux	Benthic Diatoms	g m^{-2} $year^{-1}$	Variable	PCLake simulation – year summation
Resuspension flux	Benthic Green algae	g m^{-2} $year^{-1}$	Variable	PCLake simulation – year summation
Resuspension flux	Benthic Cyanobacteria	g m^{-2} $year^{-1}$	Variable	PCLake simulation – year summation
Resuspension flux	Benthic Detritus	g m^{-2} $year^{-1}$	Variable	PCLake simulation – year summation
Assimilation eff.	Piscivorous fish	-	0.40	Supplementary Ref. 1
Assimilation eff.	Zoopl. fish (juvenile)	-	0.40	Supplementary Ref. 1
Assimilation eff.	Benth. fish (adult)	-	0.40	Supplementary Ref. 1
Assimilation eff.	Zooplankton	-	0.35	Supplementary Ref. 1
Assimilation eff.	Benthos	-	0.30	Supplementary Ref. 1
Production eff.	Piscivorous fish	-	0.17	PCLake simulation – overall average
Production eff.	Zoopl. fish (juvenile)	-	0.59	PCLake simulation – overall average
Production eff.	Benth. fish (adult)	-	0.68	PCLake simulation – overall average
Production eff.	Zooplankton	-	0.54	PCLake simulation – overall average
Production eff.	Benthos	-	0.88	PCLake simulation – overall average
Death rate	Piscivorous fish	$year^{-1}$	0.22	PCLake simulation – overall average
Death rate	Zoopl. fish (juvenile)	$year^{-1}$	4.03	PCLake simulation – overall average
Death rate	Benth. fish (adult)	$year^{-1}$	1.53	PCLake simulation – overall average
Death rate	Zooplankton	$year^{-1}$	27.3	PCLake simulation – overall average
Death rate	Benthos	$year^{-1}$	3.48	PCLake simulation – overall average
Death rate	Pelagic Diatoms	$year^{-1}$	3.66	PCLake simulation – overall average
Death rate	Pelagic Green algae	$year^{-1}$	3.66	PCLake simulation – overall average
Death rate	Pelagic Cyanobacteria	$year^{-1}$	3.66	PCLake simulation – overall average
Death rate	Benthic Diatoms	$year^{-1}$	18.3	PCLake simulation – overall average
Death rate	Benthic Green algae	$year^{-1}$	18.3	PCLake simulation – overall average
Death rate	Benthic Cyanobacteria	$year^{-1}$	73.2	PCLake simulation – overall average
Prey preference	Zoopl. -> Diatoms	-	0.75	Supplementary Ref. 1
Prey preference	Zoopl. -> Green Algae	-	0.75	Supplementary Ref. 1
Prey preference	Zoopl. -> Cyanobacteria	-	0.125	Supplementary Ref. 1
Prey preference	Zoopl. -> Detritus	-	0.25	Supplementary Ref. 1
Frac. C fixed in bones	All fish groups	-	0.35	Supplementary Ref. 1
Reproduction frac.	Benth. fish (adult)	-	0.026	PCLake simulation – overall average
Ageing fraction	Zoopl. fish (juvenile)	-	0.27	PCLake simulation – overall average

Supplementary Note 1

Differential equations, partial derivatives, and interaction strengths.

Consumers

The food web includes five groups of consumers: zoobenthos, zooplankton, piscivorous fish, juvenile benthivorous fish, and adult zooplanktivorous fish. We assume for all consumers that a fraction s of their death rate d_i is caused by density-dependent factors. The equations of zoobenthos, zooplankton, and piscivorous fish are given first, followed by the equations for juvenile benthivorous fish and adult zooplanktivorous fish, which need extra terms for the reproductive fluxes between these two groups.

Zoobenthos, zooplankton, and piscivorous fish

The differential equation of consumer group *i* is given by:

$$\frac{dX_i}{dt} = -(1-s)d_i X_i - \frac{s d_i}{X_i^*} X_i^2 - c_{i,k} X_i X_k + \sum_j a_i p_i c_{j,i} X_j X_i, \qquad (1)$$

where X_i is the biomass of consumer group i, s is the fraction of death rate d_i caused by density-dependent mortality (i.e. our stability metric), $c_{i,k}$ is the consumption coefficient of species X_i being eaten by predator X_k, a_i is the assimilation efficiency, p_i is the production efficiency, and X_j is the biomass of prey j. If group i represents the top predator piscivorous fish, then there is no predation term $-c_{i,k} X_i X_k$.

To determine the Jacobian community matrix, the partial derivative of group i to any other group j is required, evaluated in equilibrium: $\alpha_{i,j} = \left(\frac{\partial \frac{dX_i}{dt}}{\partial X_j}\right)^*$, where the star denotes equilibium.

Taking the partial derivative to predator k gives

$$\alpha_{i,k} = -c_{i,k}X_i^* = -\frac{F_{i,k}}{X_k^*}, \tag{2}$$

where $F_{i,k}$ is the feeding rate of group k on group i, given by $F_{i,k} = c_{i,k}X_i^*X_k^*$.

The partial derivative to prey j is given by

$$\alpha_{i,j} = a_i p_i X_i^* = \frac{a_i p_i F_{i,j}}{X_j^*}. \tag{3}$$

Finally, the partial derivative of consumer i to itself is given by

$$\alpha_{i,i} = -sd_i. \tag{4}$$

Juvenile (benthivorous) fish and adult (zooplanktivorous) fish

The differential equations for juvenile and adult fish are the same as for the above consumers, but include extra terms for the 'exchange' between juvenile and adult fish. Additional assumptions are:

- Adult fish lose biomass due to reproduction. This loss in adult fish biomass is added to juvenile fish biomass. The flux from adults to juveniles is independent of juvenile biomass.
- Juvenile fish lose biomass due to juveniles becoming adults. This loss in juvenile biomass is added to adult fish biomass. The flux from juveniles to adults is independent of adult biomass.
- Adult fish do not eat juveniles.

The differential equation of juvenile fish is given by

$$\frac{dX_{JF}}{dt} = -g_{JF}X_{JF} + h_{AF}X_{AF} - (1-s)d_{JF}X_{JF} - \frac{sd_{JF}}{X_{JF}^*}X_{JF}^2 - c_{JF,k}X_{JF}X_k + a_{JF}p_{JF}c_{j,JF}X_jX_{JF}, \quad (5)$$

where X_{JF} is the biomass of juvenile fish, g_{JF} is the growth rate of juvenile fish biomass turning into adult fish biomass X_{AF}, h_{AF} is the adult fish biomass loss rate due to reproduction of adults, X_k is the biomass of predator *k*, and X_j is the biomass of prey *j*.

Taking the partial derivative to adult fish gives

$$\alpha_{JF,AF} = h_{AF}. \quad (6)$$

The partial derivative to predator *k* is given by

$$\alpha_{JF,k} = -c_{JF,k}X_{JF}^* = -\frac{F_{JF,k}}{X_k^*}. \quad (7)$$

The partial derivative to prey *j* is given by

$$\alpha_{JF,j} = a_{JF}p_{JF}c_{j,JF}X_{JF}^* = \frac{a_{JF}p_{JF}F_{j,JF}}{X_j^*}. \quad (8)$$

Finally, the partial derivative of juvenile fish to itself is given by:

$$\alpha_{JF,JF} = -\frac{h_{AF}X_{AF}^*}{X_{JF}^*} - sd_{JF}X_{JF}^*. \quad (9)$$

The differential equation of adult fish is given by

$$\frac{dX_{AF}}{dt} = g_{JF}X_{JF} - h_{AF}X_{AF} - (1-s)d_{AF}X_{AF} - \frac{sd_{AF}}{X_{AF}^*}X_{AF}^2 - c_{AF,k}X_{AF}X_k + a_{AF}p_{AF}c_{j,AF}X_jX_{AF}. \quad (10)$$

Taking the partial derivative to juvenile fish gives

$$\alpha_{AF,JF} = g_{JF}. \quad (11)$$

The partial derivative to predator *k* is given by

$$\alpha_{AF,k} = -c_{AF,k}X_{AF}^* = -\frac{F_{AF,k}}{X_k^*}. \quad (12)$$

The partial derivative to prey *j* is given by

$$\alpha_{AF,j} = a_{AF} p_{AF} c_{j,AF} X_{AF}^{*} = \frac{a_{AF} p_{AF} F_{j,AF}}{X_j^{*}}. \tag{13}$$

Finally, the partial derivative of adult fish to itself is given by:

$$\alpha_{AF,AF} = -\frac{g_{JF} X_{JF}^{*}}{X_{AF}^{*}} - s d_{AF} X_{AF}^{*}. \tag{14}$$

Phytoplankton

The food web includes three types of phytoplankton: cyanobacteria, diatoms, and green algae. These three groups are present in both the water (pelagic) and the sediment (benthic) compartment. For each phytoplankton group, biomass exchange takes place between the water and sediment compartment via settling and re-suspension.

For phytoplankton, we assume that:

- Death rate of phytoplankton in both water and sediment can be split in density dependent and density independent mortality.
- The suspension and re-suspension fluxes are independent of each other, i.e. the settling flux is independent of benthic phytoplankton biomass, and the re-suspension flux is independent of pelagic phytoplankton biomass.
- Phytoplankton in the sediment do not reproduce (no growth rate *r*).

The differential equation of phytoplankton in the water compartment for group i (iW) is given by:

$$\frac{dX_{iW}}{dt} = -n_{iW} X_{iW} + m_{iS} X_{iS} + r_{iW} X_{iW} - (1-s) d_{iW} X_{iW} - \frac{s d_{iW}}{X_{iW}^{*}} X_{iW}^{2} - c_{iW,k} X_{iW} X_k, \tag{15}$$

where X_{iW} is the biomass of water phytoplankton group iW, n_{iW} is the sedimentation rate of water phytoplankton to sediment phytoplankton, m_{iS} is the re-suspension rate of sediment phytoplankton to water phytoplankton, X_{iS} is the biomass of sediment phytoplankton group i (iS), and r_{iW} is the growth rate.

If group j is sediment phytoplankton (iS), this gives

$$\alpha_{iW,iS} = m_{iS}. \tag{16}$$

If group j is a predator k of group iW, this gives

$$\alpha_{iW,k} = -c_{iW,k}X_k^* = -\frac{F_{iW,k}}{X_{iW}^*}. \tag{17}$$

Finally, the partial derivative of water phytoplankton to itself is given by

$$\alpha_{iW,iW} = -\frac{m_{iS}}{X_{iW}^*}X_{iS}^* - sd_{iW}. \tag{18}$$

The differential equation of phytoplankton in the sediment compartment for group i (iS) is given by:

$$\frac{dX_{iS}}{dt} = n_{iW}X_{iW} - m_{iS}X_{iS} + -(1-s)d_{iS}X_{iS} - \frac{sd_{iS}}{X_{iS}^*}X_{iS}^2 - c_{iS,k}X_{iS}X_k, \tag{19}$$

where X_{iS} is the biomass of sediment phytoplankton group iS, and X_k is the biomass of predator k.

Taking the partial derivative to water phytoplankton group iW gives

$$\alpha_{iS,iW} = n_{iW}. \tag{20}$$

The partial derivative to predator k is given by

$$\alpha_{iS,k} = -c_{iS,k}X_k^* = -\frac{F_{iS,k}}{X_{iS}^*}. \tag{21}$$

Finally, the partial derivative of sediment phytoplankton to itself is given by

$$\alpha_{iS,iS} = -\frac{n_{iW}}{X_{iS}^*}X_{iW}^* - sd_{iS}. \tag{22}$$

Detritus

The food web includes two groups of detritus: one in the water compartment and one in the sediment compartment. These groups are linked via suspension and re-suspension flows between the detritus pools. Assumptions for detritus are:

- Detritus receives allochthonous input (optional) (cf. Moore & De Ruiter 2012[4]).
- All biomass lost through mortality in phytoplankton and consumers, both through density-independent and density-dependent causes, is assumed to enter the detritus pools. Depending on whether the species lives in water or sediment, dead material goes to water detritus or sediment detritus, respectively. A fraction of the fish bones is removed from the system.
- The suspension and re-suspension fluxes are independent of each other, i.e. the suspension flux is independent of detritus biomass in the sediment, and the re-suspension flux is independent of detritus biomass in the water.

The differential equation of water detritus is given by

$$\frac{dX_{DW}}{dt} = R_{DW} - n_{DW}X_{DW} + m_{DS}X_{DS} + \sum_i \sum_j (1 - a_j)c_{i,j}X_iX_j + \sum_i (1 - s)d_iX_i + \sum_i \frac{sd_i}{X_i^*}X_i^2 - \sum_j c_{DW,j}X_{DW}X_j, \tag{23}$$

where X_{DW} is the biomass of water detritus, R_{DW} is allochthonous input, n_{DW} is the sedimentation rate of water detritus to sediment detritus, m_{DS} is the re-suspension rate of sediment detritus to water detritus, and X_{DS} is the biomass of sediment detritus.

Taking the partial derivative to sediment detritus, this gives

$$\alpha_{DW,DS} = m_{DS}. \tag{24}$$

The partial derivative to any phytoplankton or consumer group *j* gives

$$\alpha_{DW,j} = \sum_i (1 - a_j) c_{i,j} X_i^* + (1 - a_k) c_{j,k} X_k^* + (1 + s) d_j - c_{DW,j} X_{DW}^* = \\ = \sum_i \frac{(1 - a_j) F_{i,j}}{X_j^*} + \frac{(1 - a_k) F_{j,k}}{X_j^*} + (1 + s) d_j - \frac{F_{DW,j}}{X_j^*}, \tag{25}$$

where X_i is prey biomass eaten by *j* (this term is absent if group *j* is phytoplankton), and X_k is predator biomass consuming *j* (this term is absent if group *j* is the top predator). If group *j* does not consume water detritus, then the last term of $\alpha_{DW,j}$ is absent.

Finally, the partial derivative of water detritus to itself is given by

$$\alpha_{DW,DW} = -n_{DW} - \sum_j a_j c_{DW,j} X_j^* = -n_{DW} - \sum_j \frac{a_j F_{DW,j}}{X_{DW}^*}. \tag{26}$$

The equations for sediment detritus are very similar to equations (23)-(26) for water detritus:

$$\frac{dX_{DS}}{dt} = R_{DS} + n_{DW} X_{DW} - m_{DS} X_{DS} + \sum_i \sum_j (1 - a_j) c_{i,j} X_i X_j + \sum_i (1 - s) d_i X_i \\ + \sum_i \frac{s d_i}{X_i^*} X_i^2 - \sum_j c_{DS,j} X_{DS} X_j, \tag{27}$$

$$\alpha_{DS,DW} = n_{DW}, \tag{28}$$

$$\alpha_{DS,j} = \sum_i \frac{(1 - a_j) F_{i,j}}{X_j^*} + \frac{(1 - a_k) F_{j,k}}{X_j^*} + (1 + s) d_j - \frac{F_{DS,j}}{X_j^*}, \tag{29}$$

$$\alpha_{DS,DS} = -m_{DS} - \sum_j \frac{a_j F_{DS,j}}{X_{DS}^*}. \tag{30}$$

Supplementary References

1. Janse, J.H. *Model studies on the eutrophication of shallow lakes and ditches* (Thesis Wageningen University, 2005). Available at: http://edepot.wur.nl/121663.
2. Neutel, A.-M., Heesterbeek, J. A. P. & de Ruiter, P. C. Stability in real food webs: weak links in long loops. Science 296, 1120–1123 (2002).
3. Neutel, A.-M. et al. Reconciling complexity with stability in naturally assembling food webs. Nature 449, 599–602 (2007).
4. Moore, J. C. & de Ruiter, P. C. *Energetic Food Webs: An Analysis of Real and Model Ecosystems.* (Oxford University Press, USA, 2012)
